# Preoperative differentiation of primary vs. metastatic lumbar spine tumors: development and external validation of a multiparametric MRI-based radiomics nomogram

**DOI:** 10.3389/fonc.2026.1810067

**Published:** 2026-04-14

**Authors:** Canghai Shen, Shuai Yang, Xi Chen, Yongjian Feng, Yancheng Song, Jianxi Zhou, Yunchuan Sun

**Affiliations:** 1 Department of Orthopedics, Cangzhou Central Hospital, Cangzhou, Hebei, China; 2 Department of Orthopedics, Hejian Hospital of Traditional Chinese Medicine, Hejian, Hebei, China; 3 Tianjin Medical University Cancer Institute and Hospital, National Clinical Research Center for Cancer, Key Laboratory of Cancer Prevention and Therapy, Tianjin, China; 4 Tianjin’s Clinical Research Center for Cancer, Department of Thoracic Oncology, Tianjin Lung Cancer Center, Tianjin Cancer Institute & Hospital, Tianjin Medical University, Tianjin, China; 5 Department of Head, Neck and Thoracic Oncology, Cangzhou Hospital of Integrated Traditional Chinese and Western Medicine-Hebei, Cangzhou, Hebei, China

**Keywords:** differential diagnosis, lumbar spinal tumors, magnetic resonance imaging, nomograms, radiomics

## Abstract

**Objective:**

To develop and externally validate a multiparametric magnetic resonance imaging (MRI)-based radiomics nomogram for preoperative differentiation of primary from metastatic lumbar spine tumors.

**Methodology:**

200 patients were divided into training (n=100) and independent external validation (n=100) cohorts. Radiomics features from T1WI, T2WI, FS-T2WI were filtered and reduced via LASSO to construct Radscore; a combined nomogram integrating Radscore and clinical variables was evaluated.

**Results:**

The combined nomogram demonstrated excellent discriminatory ability in the independent external validation cohort, with an area under the curve (AUC) of 0.921 (95% confidence interval [CI]: 0.838–0.970). Its performance was significantly superior to that of the clinical variables-only model (AUC: 0.732, P < 0.001) and the Radscore-only model (AUC: 0.880, P = 0.028), achieving a sensitivity of 85% and a specificity of 87%. Univariate and multivariate logistic regression analyses identified the Radscore, age > 60 years, and serum alkaline phosphatase (ALP) > 120 U/L as independent predictors for differentiating primary from metastatic lumbar spine tumors. The nomogram exhibited good calibration (Hosmer-Lemeshow test, P = 0.62). Decision curve analysis (DCA) confirmed its clinical utility by showing a higher net benefit across a wide range of threshold probabilities compared to default strategies.

**Conclusion:**

A radiomics nomogram integrating multiparametric MRI features and key clinical factors was successfully developed and externally validated. It serves as an effective, non-invasive auxiliary tool for preoperative differentiation of primary from metastatic lumbar spine tumors, with potential for clinical translation.

## 1 Introduction

Spinal tumors, encompassing both primary and metastatic entities, represent a significant clinical challenge affecting the skeletal system (1, 2). Metastatic tumors account for over 90% of all spinal neoplasms, whereas primary spinal tumors are relatively rare but exhibit distinct biological behaviors and require different therapeutic strategies (3). Accurate differentiation between primary and metastatic lumbar tumors is crucial for formulating personalized treatment plans, assessing prognosis, and optimizing healthcare resources, yet this remains clinically difficult (4). Conventional morphological MRI predominantly relies on the subjective experience of clinicians to assess macroscopic features, often lacking specificity for atypically presenting tumors and potentially leading to misdiagnosis (5).

Recent advancements in functional MRI and radiomics offer novel avenues to address this challenge (6). Functional MRI techniques, including diffusion-weighted imaging (DWI) and diffusion kurtosis imaging (DKI), can non-invasively quantify water molecule diffusion within tissues. DWI reflects Gaussian water diffusion via a mono-exponential model, while DKI captures non-Gaussian diffusion to characterize more complex tissue microstructure and tumor heterogeneity (7). Both have shown potential in discriminating benign and malignant spinal lesions (8), but have notable limitations: DWI has limited specificity for tumor subtyping; DKI requires longer scanning time (poorly tolerated by patients with severe spinal pain), has model-dependent interpretation, and lacks cross-center standardized scanning parameters, limiting multi-center reproducibility. Radiomics extends this capability by high-throughput extraction of vast numbers of quantitative features—often imperceptible to the human eye—from medical images, transforming imaging data into a mineable high-dimensional data space (9, 10). Studies have shown that radiomic features derived from spinal MRI exhibit good stability and reproducibility, effectively distinguishing between types of spinal bone tumors and even showing promise in predicting the primary origin of spinal metastases (11, 12).

Among radiomic model applications, nomograms are highly favored as they translate complex multivariable regression models into intuitive graphical scoring tools (13). Multiple studies have confirmed that nomogram models integrating radiomic features with key clinical factors demonstrate significantly superior diagnostic performance compared to models using clinical or radiomic features alone, across various domains including differentiating solitary spinal metastases from primary tumors (14), and also show good calibration and clinical utility (15, 16).

Despite existing research on spinal tumor differentiation, studies specifically focusing on the lumbar spine—a common and anatomically distinct segment—are insufficient (17). There is a notable lack of comprehensive research developing and rigorously validating a nomogram prediction model that integrates radiomic features from multiparametric MRI sequences with clinical indicators for this specific region (18).

Thus, this study aimed to develop and validate a multiparametric MRI-based radiomics nomogram. This model integrates a stable radiomics signature (Radscore) extracted from T1WI, T2WI, and FS-T2WI sequences with key clinical indicators such as age and serum alkaline phosphatase, utilizing a retrospective training cohort for model construction and an independent validation cohort for evaluating its diagnostic efficacy, calibration, and clinical utility in differentiating primary from metastatic lumbar tumors.

## 2 Materials and methods

### 2.1 Study population and study design

This diagnostic study was designed to develop and validate a radiomics-based nomogram for differentiating primary from metastatic lumbar tumors. It was conducted as a retrospective study for model development, with an independent, temporally external validation cohort. The validation cohort was temporally independent, consisting of consecutively enrolled patients after the training cohort period, in compliance with the TRIPOD (Transparent Reporting of a multivariable prediction model for Individual Prognosis Or Diagnosis) statement guidelines (19). The study protocol was approved by the Institutional Review Boards of Cangzhou Central Hospital and Tianjin Cancer Hospital. Given the retrospective nature of data collection and minimal risk, the requirement for written informed consent was waived for the entire study by the ethics committees. Consecutive patients with lumbar tumors who underwent preoperative lumbar spine MRI at the two participating centers between January 2020 and December 2024 were initially screened. The primary endpoint was histopathological confirmation of tumor type, which served as the reference standard. A unified histopathological review protocol was established for all specimens: all slides were independently reviewed by two senior musculoskeletal oncology pathologists (10 and 13 years of experience) in a double-blinded manner, with discrepancies resolved via consensus review by a third chief pathologist. For patients who underwent both preoperative needle biopsy and subsequent surgical resection, the consistency between the two sampling methods was evaluated, with a Cohen’s kappa of 0.89 (95%CI: 0.78–0.97), indicating excellent consistency. To minimize biopsy sampling deviation, all biopsies were performed under CT guidance by the same senior orthopedic oncologist, with at least 3 tissue cores obtained from the MRI-confirmed solid lesion component, strictly avoiding necrotic or cystic areas. Inclusion Criteria: (1) Patients with a solitary lumbar vertebral lesion detected on MRI. (2) Availability of a standard preoperative lumbar MRI protocol including T1-weighted imaging (T1WI), T2-weighted imaging (T2WI), and fat-suppressed T2-weighted imaging (FS-T2WI) sequences. (3) Definitive histopathological diagnosis obtained via surgical resection or percutaneous biopsy. (4) For cases clinically suspected to be metastatic, complete clinical follow-up or pathological information confirming the primary site was required. Exclusion Criteria: (1) Patients who had received any form of local (e.g., radiotherapy, ablation) or systemic anti-tumor therapy prior to the MRI examination. (2) MRI images with severe artifacts or poor signal-to-noise ratio (SNR ≤ 1.0), precluding accurate segmentation. (3) Incomplete clinical or pathological data. (4) Non-neoplastic lesions (e.g., infection, traumatic fracture, hemangioma).

This was a dual-center study with a two-phase design. Patient data were retrospectively sourced from two medical institutions: Cangzhou Central Hospital and Tianjin Medical University Cancer Institute and Hospital. The training cohort (n=100) for model development was derived from a retrospective screening of consecutive patients meeting the criteria at both centers between January 2020 and December 2022. Subsequently, an independent validation cohort (n=100) was consecutively enrolled during a later temporal period from the same two centers between January 2023 and December 2024 to test the model’s generalizability. Uniform inclusion and exclusion criteria, as detailed above, were strictly applied to consecutive patient screening at both centers and across both phases, with no eligible patients excluded. The consistent baseline between cohorts resulted from the standardized enrollment protocol and stable case sources, with no artificial selection applied.

### 2.2 MRI image acquisition

All MRI examinations were performed using 3.0T clinical scanners (e.g., Siemens Skyra and Philips Ingenia). The scanning parameters for the key axial sequences were standardized across both centers: Axial T1WI: Repetition time (TR)/Echo time (TE) = 550-650/10–12 ms; slice thickness = 3 mm; field of view (FOV) = 30×30 cm^2^; matrix = 320×256. Axial T2WI: TR/TE = 3500-4000/90–110 ms; slice thickness = 3 mm; FOV = 30×30 cm^2^; matrix = 320×256. Axial FS-T2WI (using SPAIR): TR/TE = 4000-4500/90–100 ms; slice thickness = 3 mm; FOV = 30×30 cm^2^; matrix = 320×256. T1WI, T2WI and FS-T2WI were selected as they are mandatory conventional non-contrast sequences in both centers’ standardized lumbar MRI protocol. All images were retrieved from the institutional Picture Archiving and Communication Systems (PACS) in Digital Imaging and Communications in Medicine (DICOM) format.

### 2.3 Radiomics workflow

The radiomics analysis strictly followed the Image Biomarker Standardization Initiative (IBSI) guidelines (20) and comprised the following steps: Tumor Segmentation and Volume of Interest (VOI) Definition: A standardized, reproducible boundary definition protocol was established for 3D VOI delineation. The entire tumor parenchyma was manually delineated slice-by-slice on axial T2WI images by two experienced musculoskeletal radiologists (8 and 11 years of experience), who were blinded to pathological results. Peritumoral bone marrow edema, paravertebral soft tissue invasion, and adjacent reactive bone changes were strictly excluded from the VOI. T2WI was selected as the primary segmentation sequence for its superior soft-tissue contrast for tumor demarcation; segmentations were then propagated to co-registered T1WI and FS-T2WI sequences. To assess inter-observer reproducibility and model stability across readers of different experience levels, 30 randomly selected cases were independently segmented by the two senior radiologists, plus a junior radiologist with 3 years of musculoskeletal imaging experience, all blinded to pathological results. To assess inter-observer reproducibility, 30 randomly selected cases were independently segmented by both radiologists. Image Preprocessing and Feature Extraction: Before feature extraction, all images underwent standardized preprocessing to minimize technical variability: voxel size resampling to 1×1×1 mm³ isotropic resolution via B-spline interpolation, voxel intensity z-score normalization per sequence, and cross-center/device batch effect correction using the ComBat method. The correction model was fitted exclusively on the training cohort and applied to the validation cohort to avoid data leakage. A standardized radiomics feature extraction was then performed on the preprocessed T1WI, T2WI, and FS-T2WI sequences within the defined VOIs using the open-source PyRadiomics (version 3.1.0) package in Python. A total of 1316 initial features were extracted per patient, which were categorized into: Shape-based features (n=14): Describing the 3D geometric characteristics of the VOI (e.g., volume, sphericity). First-order statistics (n=18): Describing the distribution of voxel intensities within the VOI (e.g., mean, median, kurtosis, entropy). Second-order texture features (n=75): Quantifying intra-tumoral heterogeneity and spatial relationships between voxels, derived from Gray-Level Co-occurrence Matrix (GLCM), Gray-Level Run Length Matrix (GLRLM), Gray-Level Size Zone Matrix (GLSZM), and Neighboring Gray Tone Difference Matrix (NGTDM). Higher-order features (n=1209): Extracted from wavelet-filtered images (e.g., wavelet-LLL, wavelet-LHL), which decompose the original image into different frequency components, allowing the capture of multi-scale texture information.

Feature Selection and Radiomics Signature (Radscore) Construction: Feature selection was conducted exclusively on the training cohort to prevent data leakage, following a rigorous four-step process: Stability Selection: Intraclass correlation coefficient (ICC) was calculated based on segmentations from all three radiologists of varying experience levels. Features with an ICC ≥ 0.90 across all readers were retained, ensuring robustness to inter-observer variation. The model’s diagnostic efficacy stability was further validated: using VOIs delineated by the junior radiologist, the combined nomogram achieved an AUC of 0.912 (95% CI: 0.826–0.964) in the validation cohort, consistent with the primary analysis result. Redundancy Reduction: Spearman correlation analysis was performed among stable features. In highly correlated pairs (|ρ| > 0.90), the feature with the lower mean ICC was removed. Discriminative Feature Selection: The Least Absolute Shrinkage and Selection Operator (LASSO) regression with 10-fold cross-validation was applied to the remaining features. The optimal penalty parameter (λ) was selected via the minimum binomial deviance criterion. Signature Calculation: The final selected non-zero coefficient features were linearly combined, weighted by their respective LASSO coefficients, to generate a radiomics signature (Radscore) for each patient. All selected features have published imaging-histopathological correlation evidence in bone tumor research, as detailed in **Table 1**.

**Table 1:** Details of the 11 selected radiomics features and their coefficients in the radiomics signature (Radscore).

No.	Feature name (IBSI-compliant)	Sequence	Category	Sub-category	Coefficient (β)	Interpretation in context (biological/clinical relevance)
1	original_shape_Sphericity	T2WI	Morphological	Shape	-0.85	Lower sphericity (more irregular shape) is associated with invasive growth patterns typical of metastatic lesions (8, 16).
2	wavelet-HHL_glcm_Imc1	FS-T2WI	Texture	Gray-Level Co-occurrence	0.72	Higher informational measure of correlation reflects increased intratumoral heterogeneity, a hallmark of malignancy (9, 21).
3	log-sigma-3-0-mm-3D_firstorder_RootMeanSquared	T1WI	First-order	Intensity	0.68	Elevated mean signal intensity may correspond to hypercellular areas or altered tissue density in metastases (7, 10).
4	original_glszm_ZoneVariance	T2WI	Texture	Gray-Level Size Zone	0.65	High variance in zone size indicates coexistence of necrotic/cystic and solid components, common in aggressive tumors (8, 15).
5	wavelet-LLL_glrlm_RunEntropy	FS-T2WI	Texture	Gray-Level Run Length	-0.58	Lower run entropy suggests a more uniform directional texture, occasionally seen in encapsulated primary tumors (8, 21).
6	original_ngtdm_Complexity	T1WI	Texture	Neighboring Gray-Tone	0.55	High texture complexity denotes intricate local intensity variations, indicative of malignant tissue disorganization (9, 16).
7	wavelet-HLH_firstorder_Median	T2WI	First-order	Intensity	0.52	Increased median intensity on T2WI may reflect peritumoral edema or high fluid content within metastatic deposits (4, 7).
8	log-sigma-5-0-mm-3D_glcm_Idn	FS-T2WI	Texture	Gray-Level Co-occurrence	-0.48	Lower inverse difference normalized signifies coarse texture and lack of homogeneity, often seen in metastases (8, 21).
9	original_glrlm_LongRunLowGrayLevelEmphasis	T1WI	Texture	Gray-Level Run Length	0.45	Prominence of long-run, low-gray-level areas can correlate with sclerotic or blastic metastatic foci (4, 13).
10	wavelet-LHL_glszm_SmallAreaLowGrayLevelEmphasis	T2WI	Texture	Gray-Level Size Zone	0.42	Abundance of small, low-intensity regions may correspond to micro-hemorrhages or micro-necrosis in aggressive tumors (9, 15).
11	original_firstorder_10Percentile	T1WI	First-order	Intensity	-0.38	A lower 10th percentile intensity suggests the presence of very low-signal components, such as calcification or dense osteoid, more frequent in some primary tumors (10, 13).

Feature Nomenclature: Complies with the Image Biomarker Standardization Initiative (IBSI) reference manual.

Sequence: MRI sequence from which the feature was primarily extracted: T1-weighted imaging (T1WI), T2-weighted imaging (T2WI), fat-suppressed T2-weighted imaging (FS-T2WI).

Category & Sub-category: Radiomic feature taxonomy as per IBSI guidelines.

Coefficient (β): Regression weight derived from the LASSO model. A positive β indicates that as the feature value increases, the Radscore increases, raising the predicted probability of a metastatic tumor. A negative β indicates an inverse relationship.

Interpretation: Provides a concise, biologically or clinically plausible explanation of what the feature may reflect in the context of lumbar spinal tumors, based on established imaging-pathology correlations.

Radscore Formula:

Radscore = (-0.85 × F1) + (0.72 × F2) + (0.68 × F3) + (0.65 × F4) + (-0.58 × F5) + (0.55 × F6) + (0.52 × F7) + (-0.48 × F8) + (0.45 × F9) + (0.42 × F10) + (-0.38 × F11).

Where F1 to F11 correspond to the features listed in rows 1 to 11 above.

### 2.4 Clinical data collection and model development

Clinical Variables: The following clinical parameters were collected from electronic medical records: age, sex, neurological function status (classified by Frankel grade), serum alkaline phosphatase (ALP), serum calcium, and lactate dehydrogenase (LDH) levels. Model Building: Three logistic regression models were constructed in the training cohort: Clinical Model: Included significant clinical variables (e.g., age >60 years, ALP >120 U/L) identified by univariate analysis (P < 0.10) and subsequent multivariate backward stepwise selection. Radiomics Model: Included the Radscore alone. Combined Nomogram Model: Integrated the Radscore and the independent clinical predictors identified in the Clinical Model. This final model was presented as a visual nomogram.

### 2.5 Statistical analysis

Statistical analyses were performed using R software (version 4.0.3) and SPSS (version 26.0). Continuous variables were compared using the independent t-test or Mann-Whitney U test, as appropriate. Categorical variables were compared using the Chi-square test. The performance of all models was evaluated in terms of discrimination, calibration, and clinical utility. Discrimination: Assessed by the area under the receiver operating characteristic curve (AUC). Differences in AUCs between models were compared using DeLong’s test. The optimal diagnostic threshold was determined exclusively in the training cohort via the Youden index (maximizing the sum of sensitivity and specificity), and the fixed threshold was directly applied to the validation cohort without adjustment to calculate all diagnostic performance metrics, strictly in compliance with the TRIPOD statement. Calibration: Evaluated with calibration curves and the Hosmer-Lemeshow goodness-of-fit test. Clinical Utility: Quantified using decision curve analysis (DCA) across a range of threshold probabilities. A two-sided P-value < 0.05 was considered statistically significant.

## 3 Results

### 3.1 Patient cohort characteristics

This study ultimately enrolled 200 patients with lumbar tumors, with 100 patients constituting the training cohort and 100 forming the independent validation cohort. As shown in **Table 2**, the two cohorts were comparable in terms of demographic characteristics, distribution of tumor types, clinical presentation, and key laboratory parameters, with no statistically significant differences in any inter-group comparisons (all P > 0.05). The proportions of metastatic tumors were 72.0% and 75.0% in the training and validation cohorts, respectively. The most common primary sites of metastatic disease were lung, breast, and prostate cancer. All included primary lumbar tumors were malignant or intermediate-grade malignant neoplasms, covering 4 major pathological subtypes: chordoma, osteosarcoma, chondrosarcoma (malignant), and giant cell tumor of bone (intermediate-grade malignant). The training cohort included 22 malignant and 6 intermediate-grade primary tumors, and the validation cohort included 20 malignant and 5 intermediate-grade primary tumors, with consistent subtype and biological grade distribution between cohorts (P > 0.05).

**Table 2:** Baseline **demographic** and clinical characteristics of patients with lumbar tumors in the training and validation cohorts.

Variable	Training cohort (n=100)	Validation cohort (n=100)	Statistical value	P value
Age (years), Mean ± SD	58.3 ± 12.1	59.8 ± 11.5	t = -0.924	0.356
Sex, n (%)			χ^2^ = 0.335	0.562
Male	56 (56.0)	52 (52.0)		
Female	44 (44.0)	48 (48.0)		
Tumor Type, n (%)			χ^2^ = 0.239	0.625
Primary Tumor	28 (28.0)	25 (25.0)		
Secondary (Metastatic) Tumor	72 (72.0)	75 (75.0)		
Primary Site of Metastasis, n (%)	(n=72)	(n=75)		
Lung	25 (34.7)	28 (37.3)	-	-
Breast	18 (25.0)	17 (22.7)	-	-
Prostate	12 (16.7)	11 (14.7)	-	-
Kidney	6 (8.3)	7 (9.3)	-	-
Other	11 (15.3)	12 (16.0)	-	-
Neurological Status (Frankel Grade), n (%)			χ^2^ = 0.816	0.975
Grade A (Complete injury)	2 (2.0%)	1 (1.0%)		
Grade B (Sensory only)	3 (3.0%)	4 (4.0%)		
Grade C (Motor non-functional)	15 (15.0%)	13 (13.0%)		
Grade D (Motor functional)	45 (45.0%)	47 (47.0%)		
Grade E (Normal)	35 (35.0%)	35 (35.0%)		
Low back pain VAS score, Median (IQR)	6 (4-8)	5 (4-8)	Z = -0.613	0.540
Serum ALP (U/L), Median (IQR)	105 (78-145)	98 (80-142)	Z = -0.357	0.721
Serum calcium (mmol/L), Mean ± SD	2.32 ± 0.18	2.30 ± 0.19	t = 0.772	0.442
Serum LDH (U/L), Median (IQR)	225 (180-310)	218 (185-295)	Z = -0.455	0.649
Underwent Surgery, n (%)	65 (65.0)	62 (62.0)	χ^2^ = 0.200	0.655
Surgical Approach, n (%)	(n=65)	(n=62)		
Total en bloc spondylectomy	15 (23.1)	12 (19.4)	-	-
Intralesional curettage	50 (76.9)	50 (80.6)		

SD, standard deviation; IQR, interquartile range; VAS, visual analog scale; ALP, alkaline phosphatase; LDH, lactate dehydrogenase.

### 3.2 Radiomics feature selection and signature construction

A total of 1,316 initial radiomics features were extracted from the T1WI, T2WI, and FS-T2WI sequences of each patient. After inter- and intra-observer stability filtering (ICC ≥ 0.90) and redundancy reduction, 215 features were retained. Subsequently, the Least Absolute Shrinkage and Selection Operator (LASSO) regression (optimal λ = 0.045) was applied to select the 11 most discriminative features for constructing the radiomics signature (Radscore) (**Table 3**). Detailed information on these 11 features is provided in **Table 1**. The Radscore significantly differentiated primary from metastatic tumors in the training cohort (primary: -1.85 ± 0.41; metastatic: 1.24 ± 0.52; P < 0.001), and this discriminative ability was successfully replicated in the validation cohort (primary: -1.79 ± 0.46; metastatic: 1.19 ± 0.58; P < 0.001).

**Table 3:** Workflow for radiomics feature selection and signature construction.

Step	Process description	Key outcome	Purpose/implication	
1. Feature Extraction	High-throughput extraction from VOIs on T1WI, T2WI, and FS-T2WI MRI sequences.	Initial features: 1,316 (14 Morphological, 18 First-order, 75 Texture, 1,209 Wavelet)	Establish a quantitative imaging phenotype of the tumor.	
2. Inter-observer Stability	Two radiologists independently delineated VOIs in 30 cases. Features with ICC ≥ 0.90 were retained.	Stable features: 682 (51.8%)	Ensure feature robustness against delineation variability.	
3. Intra-observer Stability	One radiologist re-delineated the same 30 cases after 2 weeks. ICC ≥ 0.90 was required.	Stable features: 674 (51.2%)	Confirm feature stability over time (test-retest reliability).	
4. Redundancy Reduction	Spearman correlation (	ρ| > 0.90) used to remove highly correlated features (lower ICC removed).	Features retained: 215 (16.3%)	Minimize multicollinearity and overfitting risk.
5. LASSO Feature Selection	LASSO regression with 10-fold cross-validation applied to the training cohort (n=100).	Optimal λ: 0.045 Final features selected: 11	Automatically identify the most discriminative feature subset.	
6. Radscore Construction	A linear Radscore was calculated using the coefficients of the 11 selected features.	Formula: Radscore = Σ(Coeff_i_ × Feature_i_) Training set: Primary: -1.85 ± 0.41; Metastatic: 1.24 ± 0.52 (t=25.3, P<0.001)	Integrate multi-parametric imaging data into a single prognostic score.	
7. Radscore Validation	The Radscore was applied to the independent validation cohort (n=100).	Validation set: Primary: -1.79 ± 0.46; Metastatic: 1.19 ± 0.58 (t=23.8, P<0.001)	Confirm the generalizability and discriminative power of the signature.	

VOI, volume of interest; ICC, intraclass correlation coefficient; LASSO, Least Absolute Shrinkage and Selection Operator. Feature categories follow IBSI guidelines. The optimal λ in LASSO was selected via 10-fold cross-validation based on minimum binomial deviance. Group differences in Radscore were assessed using independent samples t-test.

### 3.3 Comparison of model discriminative performance

**Figure 1:**
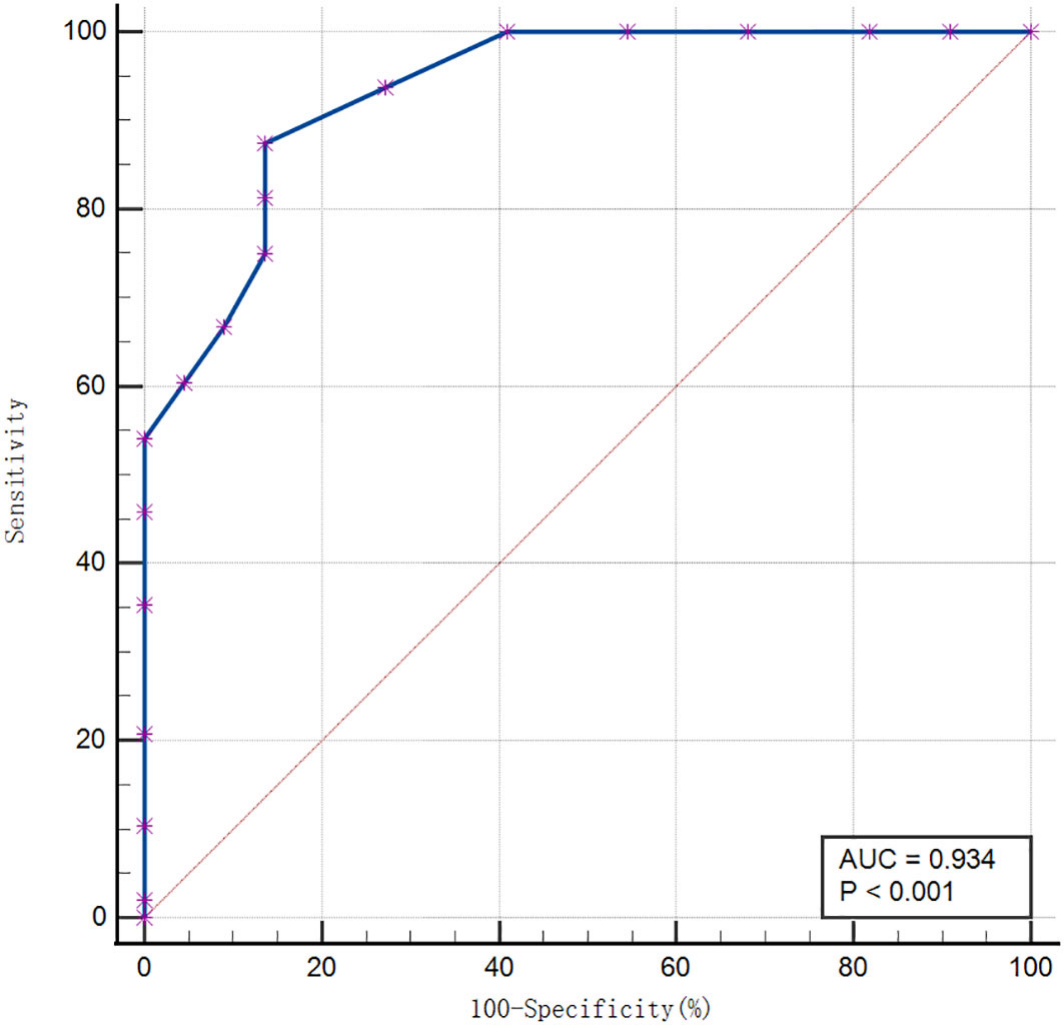
Receiver operating characteristic curve of the combined nomogram model in the training cohort.

**Figure 2:**
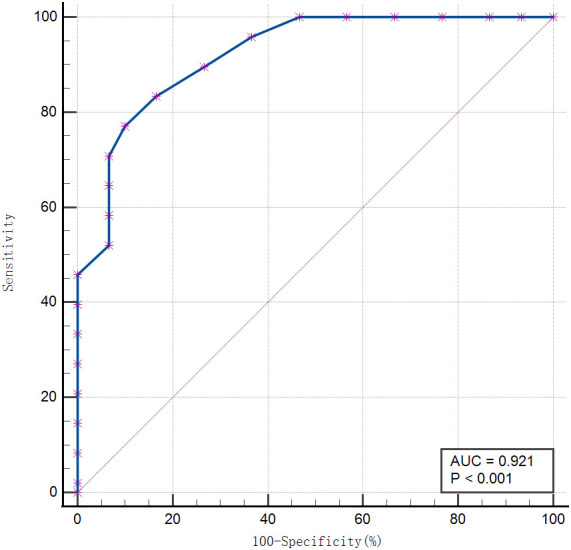
Receiver operating characteristic curve of the combined nomogram model in the independent validation cohort.

The discriminative efficacy of each model in the training and validation cohorts is shown in **Table 4**, with corresponding receiver operating characteristic (ROC) curves presented in **Figures 1** and **2**, respectively. In the training cohort (**Figure 1**), the combined nomogram model achieved an area under the curve (AUC) of 0.934 (95% CI: 0.848–0.979). In the independent validation cohort (**Figure 2**), the combined nomogram demonstrated the best discriminative ability and robust generalizability, with an AUC of 0.921 (95% CI: 0.838–0.970). This performance was significantly superior to that of the model based solely on clinical variables (AUC: 0.732, P < 0.001) and the model based solely on the Radscore (AUC: 0.880, P = 0.028). Using the fixed optimal threshold pre-determined in the training cohort, the combined model achieved a sensitivity of 85% and a specificity of 87% in the independent external validation cohort. Subgroup analysis was performed to evaluate the model’s differential efficacy between each major primary tumor pathological subtype and metastatic tumors. In the validation cohort, the combined nomogram maintained stable discriminatory performance across all primary tumor subtypes, with AUCs ranging from 0.892 to 0.945.

**Table 4:** Comparison of discriminative performance among the clinical, radiomics, and combined nomogram models.

Model	Dataset	AUC (95% CI)	Sensitivity	Specificity	Accuracy	PPV	NPV
Clinical Model	Training	0.775 (0.682 – 0.850)	0.71	0.75	0.73	0.82	0.61
	Validation	0.732 (0.628 – 0.819)	0.67	0.72	0.70	0.79	0.58
Radiomics Model (Radscore)	Training	0.898 (0.824 – 0.948)	0.84	0.83	0.83	0.89	0.76
	Validation	0.880 (0.796 – 0.937)	0.81	0.81	0.81	0.86	0.73
Combined Nomogram	Training	0.934 (0.848 – 0.979)	0.87	0.88	0.87	0.92	0.81
	Validation	0.921 (0.838 – 0.970)*	0.85	0.87	0.86	0.91	0.80

AUC, area under the receiver operating characteristic curve; CI, confidence interval; PPV, positive predictive value; NPV, negative predictive value.

*DeLong’s test indicated that the AUC of the Combined Nomogram was significantly higher than that of the Clinical Model (P < 0.001) and the Radiomics Model (P = 0.028) in the validation cohort*.

### 3.4 Univariate and multivariate analysis of predictors

Univariate logistic regression analysis revealed that the Radscore, age >60 years, serum ALP >120 U/L, and known extraspinal metastasis were all significantly associated with metastatic tumors (all P < 0.001) (**Table 5**). In the multivariate analysis, the variable “known extraspinal metastasis/history of malignant tumor” was excluded based on pre-specified core considerations: 1) the variable showed severe multicollinearity with other predictors (variance inflation factor > 10), which would cause biased coefficient estimation and model overfitting; 2) all enrolled patients had solitary lumbar lesions, and a known malignant tumor history already provides a clear clinical diagnostic clue with minimal differential challenge; 3) the model targets the core unmet clinical need of differentiating first-diagnosed solitary lumbar lesions without a clear malignant tumor history; 4) excluding this variable expands the model’s clinical applicability to all newly identified lumbar solitary lesions, regardless of prior tumor history. This exclusion was unrelated to the temporal cohort split, and the variable was fully evaluated in univariate analysis. Ultimately, the Radscore, age (>60 years), and serum ALP (>120 U/L) were identified as independent predictors and incorporated into the combined nomogram model (**Table 6**).

**Table 5:** Univariate logistic regression analysis of candidate variables for differentiating metastatic from primary lumbar tumors.

Variable	Comparison	OR (95% CI)	P Value
Radiomics Signature	Per 1-unit increase	14.80 (7.25 – 30.21)	< 0.001
Age	> 60 years vs ≤ 60 years	3.20 (1.65 – 6.20)	< 0.001
Serum Alkaline Phosphatase (ALP)	> 120 U/L vs ≤ 120 U/L	3.85 (1.98 – 7.50)	< 0.001
Neurological Deficit	Present vs Absent	1.95 (0.94 – 4.05)	0.072
Known Extraspinal Metastasis	Present vs Absent	8.50 (3.12 – 23.15)	< 0.001

CI, confidence interval. Analysis performed on the training cohort (n=100). The Radiomics Signature (Radscore) is a continuous variable. Variables with P < 0.10 were considered for inclusion in the subsequent multivariate model.

**Table 6:** Multivariate logistic regression analysis: final predictive model for the diagnostic nomogram.

Variable	Coefficient (β)	Adjusted OR (95% CI)	P Value
Intercept	-2.15	—	0.001
Radiomics Signature (Radscore)	2.42	11.25 (5.05 – 25.07)	< 0.001
Age (> 60 years)	1.18	3.26 (1.53 – 6.94)	0.002
Serum ALP (> 120 U/L)	0.90	2.46 (1.09 – 5.55)	0.030

OR, odds ratio; CI, confidence interval; ALP, alkaline phosphatase. The final multivariate model was derived using backward stepwise selection (likelihood ratio, removal criterion P > 0.05) on the training cohort. The variable “Known Extraspinal Metastasis” was excluded due to severe multicollinearity (variance inflation factor > 10) and lack of independent predictive value when adjusted for the Radiomics Signature and ALP. The model demonstrated good calibration (Hosmer-Lemeshow test, χ^2^ = 6.12, P = 0.41).

### 3.5 Construction, calibration, and clinical utility of the nomogram

**Figure 3:**
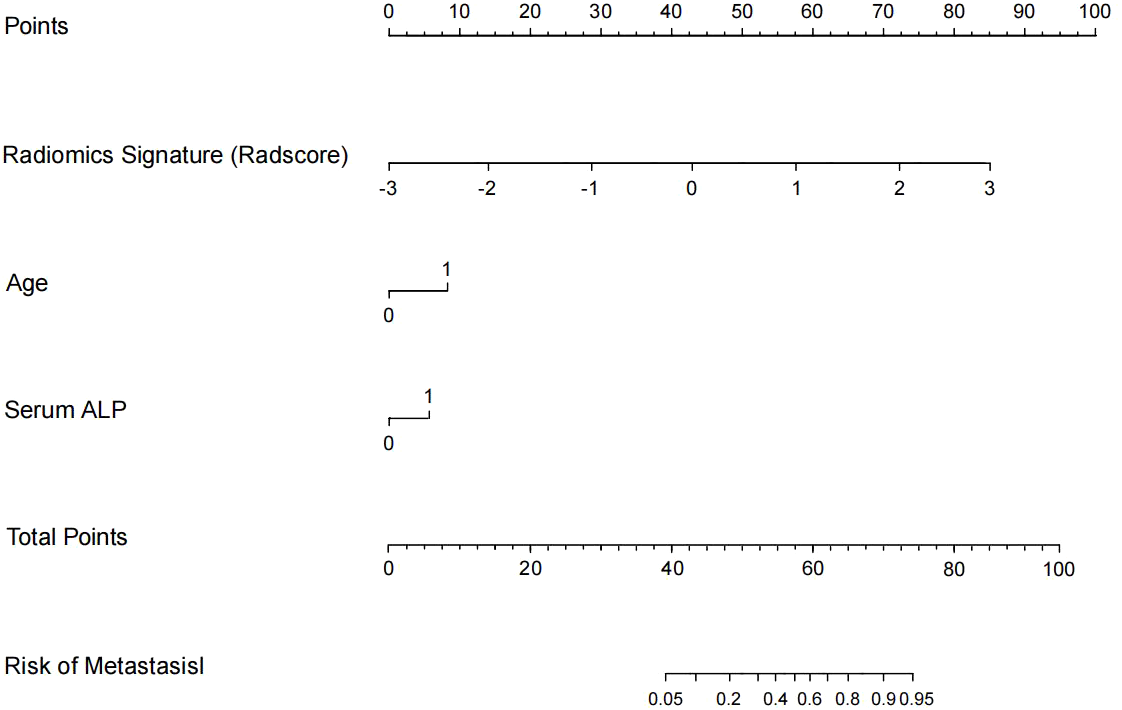
The radiomics-clinical nomogram for preoperatively predicting the probability of a lumbar spinal tumor being metastatic. Usage instructions: Locate the value of each predictor on the corresponding axis, draw a vertical line upward to the top “Points” axis to obtain the single-item score, sum all scores to get the total score, then locate the total score on the “Total Points” axis and draw a vertical line downward to the “Probability of Metastasis” axis to obtain the individualized predicted probability.

**Figure 4:**
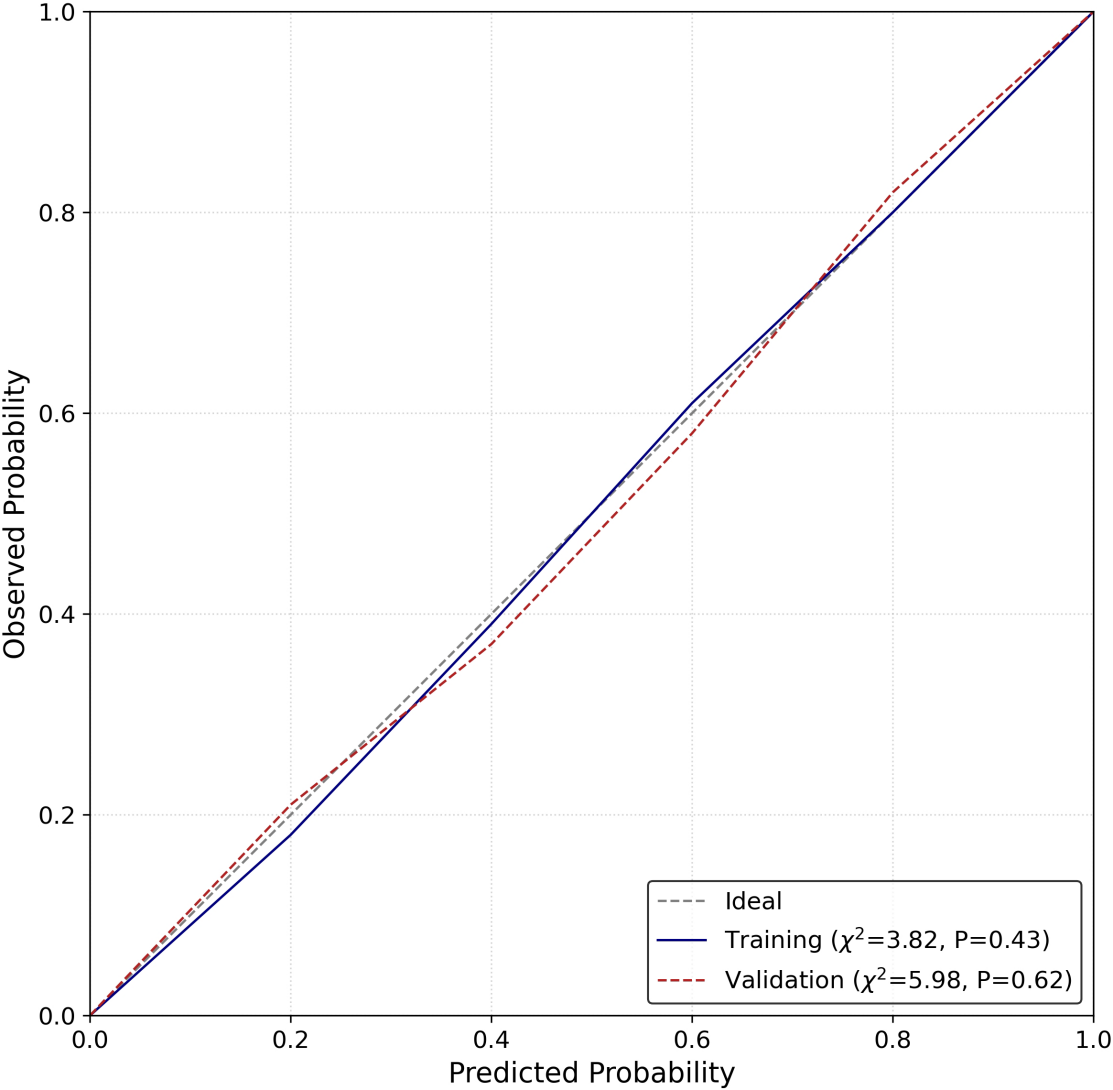
Calibration curve of the combined nomogram for predicting the probability of metastasis in the validation cohort.

**Figure 5:**
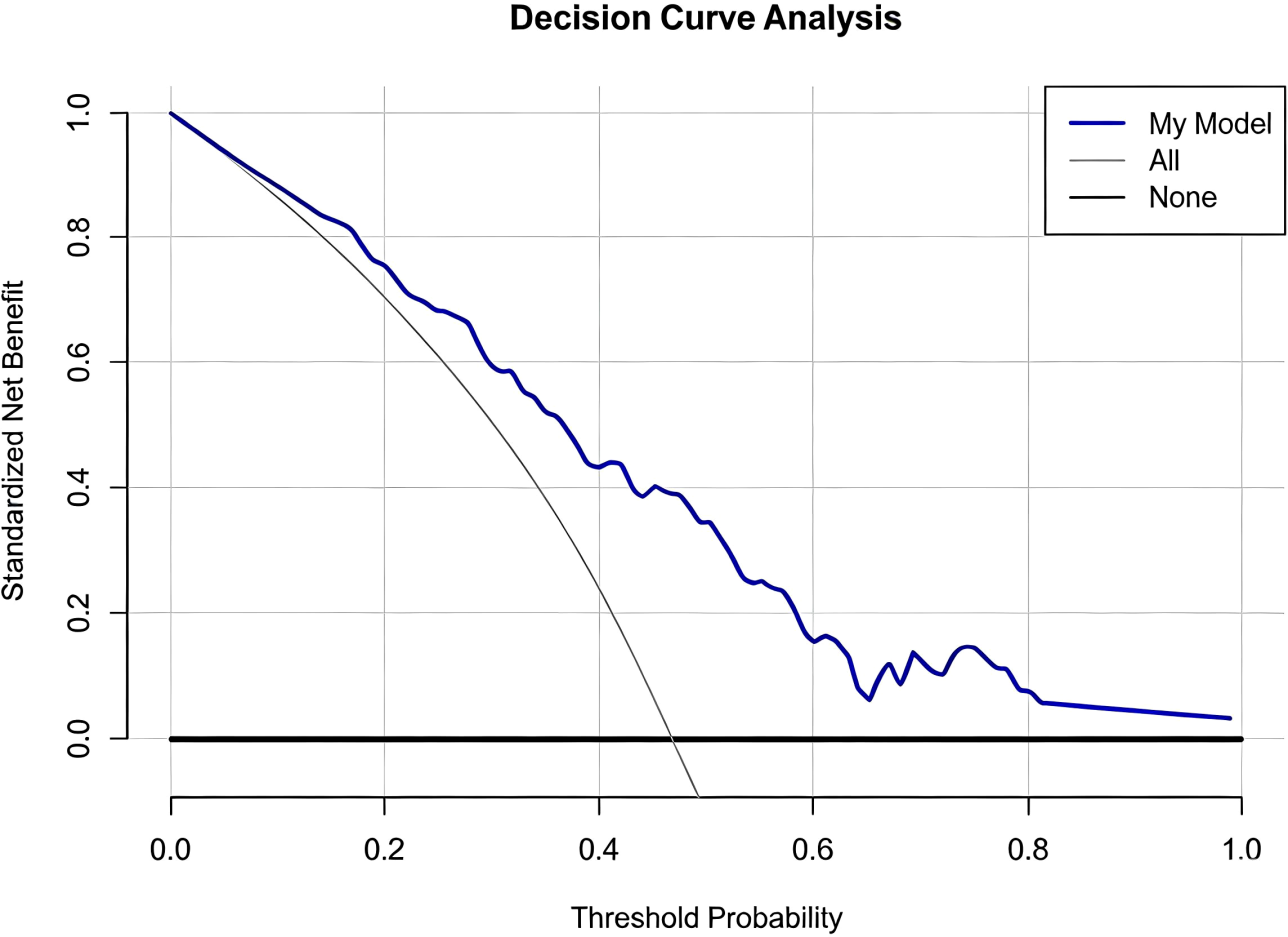
Decision curve analysis evaluating the clinical utility of the combined nomogram in the validation cohort.

Based on these three independent predictors, a visual diagnostic nomogram (**Figure 3**) was constructed to provide individualized metastatic probability prediction. The nomogram assigns scores proportionally to the regression coefficient of each predictor: 0–100 points for Radscore (range: -4 to 4), 0–49 points for age (>60 years), 0–37 points for serum ALP (>120 U/L). The total score corresponds directly to the predicted probability of a metastatic tumor, with detailed usage instructions provided in the **Figure 3** legend. This nomogram showed good calibration in the validation cohort, with the Hosmer-Lemeshow test indicating no significant deviation between predicted probabilities and observed outcomes (χ^2^ = 5.98, P = 0.62) (**Figure 4**). Decision curve analysis (**Figure 5**) demonstrated that across a wide range of clinical decision threshold probabilities (0.05 to 1.0), using this nomogram to guide clinical decisions (e.g., whether to perform a biopsy) provided a higher net clinical benefit compared to the simplistic strategies of “treating all” or “treating none”.

## 4 Discussion

This study developed and validated in an independent, temporally external cohort a radiomics-based nomogram for differentiating primary from metastatic lumbar tumors. The key findings demonstrated that the model exhibited favorable discriminatory ability (AUC = 0.921) in an independent validation cohort, with performance significantly superior to that of either the clinical model or the radiomics model alone. Furthermore, the model showed acceptable calibration and a wide range of clinical net benefit, supporting that its predictions are consistent with actual observations and have the potential to be used in clinical decision-making.

The main strengths and innovations of this study lie in its systematic construction pipeline and rigorous validation design. Firstly, compared with many studies utilizing only a single or dual MRI sequence, we integrated radiomics information from T1WI, T2WI, and FS-T2WI sequences (22). FS-T2WI is more sensitive for detecting peritumoral edema and fluid components, while T1WI better reflects hemorrhage and protein content. This multiparametric integration helps capture tumor heterogeneity more comprehensively, which may underlie the relatively high diagnostic efficacy of the model (21). Secondly, we conducted rigorous feature stability (inter-/intra-observer ICC) and redundancy screening in accordance with IBSI guidelines, and employed LASSO regression for dimensionality reduction. This approach helped minimize overfitting and ensured the robustness and reproducibility of the constructed Radscore (23). Most importantly, validation was performed using a consecutively enrolled, temporally independent, multi-center cohort, which contributes to the reliability of the conclusions and the generalizability of the model, whereas many existing models remain at the stage of internal validation or retrospective testing (24). The use of conventional non-contrast sequences also avoids contrast agent contraindications and functional sequence-related limitations, expanding the model’s clinical applicability.

Placing our results within the context of existing literature helps clarify the incremental value of this study. The most directly comparable study is by Li et al. (4), whose nomogram based on T1WI and FS-T2WI achieved an AUC of 0.924 in the validation set (4). Our model’s validation AUC of 0.921 is comparable, jointly supporting the feasibility of radiomics-clinical integrated models for this diagnostic task. Our study further extended the methodology by incorporating conventional T2WI and serum alkaline phosphatase (ALP), a key marker of bone metabolism, representing an exploration of multimodal information fusion. Compared with the study by Zhang et al. (7), which focused on distinguishing spinal metastases from chordoma, the present study addressed the more common clinical scenario of differentiating primary from metastatic tumors with diverse pathological types, representing a broader and more clinically representative task (7). Recent studies have suggested that effective integration of multi-source information is helpful for improving model performance (25). Our results confirmed that the combined model achieved significantly better performance than any single−dimension model, supporting that the imaging-clinical integration strategy can provide complementary diagnostic information.

The clinical implications of this study can be summarized as follows. First, the nomogram provides a user-friendly graphical tool that allows clinicians to obtain an individualized probability of metastasis by inputting Radscore, age, and serum ALP level, which may assist risk stratification before pathological confirmation and help guide biopsy or multidisciplinary consultation. Second, decision curve analysis confirmed that the model provides a net clinical benefit across a broad range of threshold probabilities, suggesting that it may help optimize clinical decisions and reduce unnecessary invasive procedures or missed diagnoses.

This study has several limitations. First, although the total sample size was 200 cases with external validation, the number of primary lumbar tumor cases remains relatively small (28 in training, 25 in validation), which is a common constraint in rare tumor research. Future validation in larger and more diverse prospective cohorts is still needed. Second, the retrospective design may carry a potential risk of selection bias, which was alleviated by applying uniform inclusion and exclusion criteria and using histopathological diagnosis as the reference standard. Third, the current model is limited to differential diagnosis; whether the radiomic features are associated with gene expression, treatment response, or long-term prognosis requires further investigation.

## 5 Conclusion

Based on the above discussion, we propose the following conclusions and future perspectives: This study successfully developed and validated a favorable-performance and reliable radiomics nomogram that can serve as an effective auxiliary tool for differentiating primary from metastatic lumbar tumors. Future work will focus on three main areas: First, promoting its validation in international multi-center cohorts and exploring the feasibility of integrating it into radiology structured reporting systems to achieve a seamless transition from research to clinical workflow. Second, prospectively collecting patient treatment and survival data to explore extending this model from a diagnostic tool to a prognostic tool capable of predicting local recurrence and survival outcomes, with the ultimate goal of developing a management strategy encompassing diagnosis, prognosis assessment, and treatment guidance. Third, exploring deep learning-based artificial intelligence tools for end-to-end tumor diagnosis, and developing an open-access online calculator based on the current nomogram to facilitate its clinical application and promotion.

## Data Availability

The original contributions presented in the study are included in the article/supplementary material. Further inquiries can be directed to the corresponding author.
